# Sociodemographic factors, parental mental health and movement behaviours in the early years: the SUNRISE Finland study protocol

**DOI:** 10.1186/s44167-023-00042-4

**Published:** 2024-01-03

**Authors:** Elina Engberg, Amanda Ojala, Hanna Paasio, Jari Lahti, Pasi Koski, Katri Vehviläinen-Julkunen, Raija Korpelainen, Soile Puhakka, Anthony Okely, Eva Roos

**Affiliations:** 1 Folkhälsan Research Center, Topeliuksenkatu 20, Helsinki, 00250 Finland; 2 Department of Psychology and Logopedics, University of Helsinki, Helsinki, Finland; 3 Department of Teacher Education, University of Turku, Rauma, Finland; 4 Department of Nursing Science, University of Eastern Finland, Kuopio, Finland; 5 Kuopio University Hospital, Kuopio, Finland; 6 Department of Sports and Exercise Medicine, Oulu Deaconess Institute Foundation sr, Oulu, Finland; 7 Research Unit of Population Health, Faculty of Medicine, University of Oulu, Oulu, Finland; 8 Medical Research Center, Oulu University Hospital and University of Oulu, Oulu, Finland; 9 Early Start, School of Health and Society, Faculty of the Arts, Social Sciences and Humanities, University of Wollongong, Wollongong, NSW Australia; 10 Department of Sport, Food and Natural Sciences, Western Norway University of Applied Sciences, Bergen, Norway; 11 Department of Public Health, University of Helsinki, Helsinki, Finland; 12 Department of Food Studies, Nutrition and Dietetics, Uppsala University, Uppsala, Sweden

**Keywords:** Children, Preschool, Physical activity, Sedentary behaviour, Sleep, Screen time, Motor skills, Cognitive skills, Sociodemographics, Mental health

## Abstract

**Background:**

The World Health Organization (WHO) has identified the prevention of obesity in young children as one of its key priorities for the 21st century, and 24-hour movement behaviours (physical activity, sedentary behaviour and sleep) play a key role in this priority. The SUNRISE Finland Study is part of the international SUNRISE Study, which examines the movement behaviours of young children in 64 low-, middle- and high-income countries. The SUNRISE Finland Study will investigate what proportion of 3- to 4-year-old children living in Finland meet the WHO global guidelines on 24-hour movement behaviours, and how that proportion and children’s motor and cognitive skills compare with children from other countries involved in the SUNRISE Study. We also aim to identify potential correlates of children’s movement behaviours, focusing on socioeconomic factors, residential environmental features, and parents’ mental health. In addition, this study will examine the associations between children’s movement behaviours, motor and cognitive skills, adiposity, and psychosocial wellbeing. Finally, we aim to establish a cohort of families who participate in the study and conduct follow-ups in the future.

**Methods:**

We will recruit 1,000 children aged 3.0 to 4.9 years and their caregivers through early childhood education and care centres in Finland (50% in urban and 50% in rural areas). We will assess children’s 24-h movement behaviours using two accelerometers and a parental report. Children will perform validated tests to measure gross and fine motor skills and executive functions, and their height, weight and waist circumference will be measured. Caregivers will complete questionnaires regarding sociodemographic factors, nature visits, their own movement behaviours, symptoms of depression, anxiety, stress, insomnia, nomophobia, social media self-control failure, and happiness, and child’s psychosocial wellbeing. Geographic Information System (GIS) will be used to examine residential environmental features.

**Discussion:**

In addition to facilitating international comparisons on movement behaviours and motor and cognitive skills, the SUNRISE Finland Study will provide novel evidence on factors associated with movement behaviours in young children. The results of this study will help in planning actions to promote healthy levels of movement behaviours at an early age and equal opportunities for healthy development.

**Clinical Trial Number:**

This is not a trial study.

## Background

The World Health Organization (WHO) has identified the prevention of obesity in young children as one of its key priorities for the 21st Century [1]. Movement behaviours, i.e. physical activity (PA), sedentary behaviour and sleep, play a key role in contributing to this priority. Consequently, WHO released *Guidelines on physical activity, sedentary behaviour and sleep for children under 5 years of age* in 2019. For children aged 3 to 4 years, the guidelines are over 24 h: (i) at least 180 min of PA, of which at least 60 min is moderate- to vigorous-intensity activity, (ii) no more than 1 h of sedentary screen time, and no more than 1 h of restrained sitting at a time, and (iii) 10 to 13 h of good quality sleep [2]. The early years of life (ages birth to 5) is a critical and sensitive period for the development of physical, motor, cognitive and social skills. During these early years, a child’s development and behaviours can be influenced in a positive or in a negative way.

Specific types of PA, total PA, and PA of at least moderate- to vigorous-intensity have been favourably associated with health indicators, such as motor development, fitness, and bone and skeletal health in preschool-aged children [3]. The most favourable frequency and duration of PA is, however, unclear [3]. Moreover, the scientific evidence on the associations between sedentary behaviour and health indicators in young children remain limited and equivocal [4]. These limited findings support the importance of minimizing screen time for health promotion in the early years, but also highlight the potential cognitive benefits of interactive non-screen-based sedentary behaviours. Also, studies examining whether movement behaviours relate to psychosocial wellbeing among preschool-aged children remain scarce [5–7]. Additional high-quality research using valid measures is needed to establish the relationships between durations, patterns, and types of sedentary behaviours and health indicators in the early years. Moreover, limited evidence indicates that the most ideal combinations of movement behaviours (e.g., adequate sleep, low amounts of sedentary behaviour and high levels of PA) may be important for optimal health of young children [8]. Thus, further research is needed to determine the ideal distribution of daily movement behaviours for health throughout the early years.

Ecological Systems Theory emphasizes the importance of studying children in multiple environments, such as family, day care, community, and society at large, also called socioecological systems or levels, to understand their development and behaviour [9]. This theory suggests that in addition to concentrating only on individual factors impacting development and behaviours, intrafamiliar factors, and how the intrafamiliar processes are affected by extrafamiliar conditions, should be examined. The determinants of childhood obesity and related behaviours, for example, involve factors from multiple contexts [10]. However, more specific factors related to movement behaviours in young children differ across behaviours and are not well-established [11–16].

The scientific evidence on the differences in PA between rural and urban children is sparse and shows conflicting findings [17]. One recent study showed that rural 10- to 11-year-old children spent less time being sedentary and more time in light PA than their urban counterparts [18]. Otherwise, light PA is seldom reported in studies examining urban/rural differences. Moreover, only a limited number of studies have used Geographic Information System (GIS) and accelerometers to examine the relationship between PA and built and natural environments among children [19]. The existing studies suggest that roads and streets, school grounds, and the home location are important locations for total and moderate-to-vigorous intensity PA, and possibly for light PA, among 5- to 18-year-old children and adolescents. The relationship between PA and greenspace seems positive, and domestic gardens may be important for children engaging in higher intensity PA [19]. However, further studies examining children under five years of age, as well as assessing sedentary behaviour and sleep in addition to PA are needed. The relationship between early childhood education and care (ECEC) centres’ surrounding environments and children’s movement behaviours, and how residential environment is associated with children playing in nature also warrants further investigation. The associations between PA levels in young children and their parents seem to be weak [20], suggesting that effective ways, including environmental, to promote parents and children to be active together need to be examined.

Interventions to prevent childhood obesity have mainly focused on children’s individual-level behaviour change, with limited effects [21]. Parents and family play an important role in children’s lives, particularly during their early years. Thus, some interventions designed to modify health behaviours within the families of preschoolers have targeted parental knowledge of those behaviors, role modeling, and parents’ own health behaviours [22–24]. Mental health disorders are among the major global health challenges and their contribution is rising [25]. WHO predicts that depression will be the leading cause of disease burden by the year 2030 [26]. Parental mental health may contribute to a child’s health behaviours in complex ways, but examining this relationship has received insufficient attention so far. Parents who exhibit symptoms of stress or depression may, for example, have difficulties with establishing regular schedules for sleep, screen time and PA for both the parent and the child [27]. In fact, maternal stress or depressive symptoms seem to associate with obesity, lower levels of PA, higher levels of sedentary behaviours, and unhealthy dietary behaviours among their young children, but evidence is limited and shows mixed findings [13, 27–29]. Furthermore, we previously showed that greater parental happiness is associated with preschool-aged children engaging in multiple healthy behaviours [30]. The majority of previous studies used maternal reports of children’s behaviours and did not assess movement behaviours with a more objective method, such as an accelerometer.

Because targeting parental knowledge of healthy behaviours and role modeling may not be effective enough [23], further studies examining other family-related determinants, such as parents’ mental wellbeing are needed. Other parental factors, such as higher maternal body mass index, lower PA levels and high screen time have been associated with their children engaging in lower levels of PA and higher amounts of sedentary behaviours in some studies, whereas results on the association between parental education or income and preschool-aged children’s movement behaviours are mixed [11–16]. Previous studies examining family-related determinants of children’s movement behaviours have mostly included one parent and usually the mother. Further research is necessary to explore environmental, socioeconomic and behavioural determinants of movement behaviours that are under-examined in this population. Because of the limited scientific evidence so far, it is difficult to draw conclusions on the most important factors to target in interventions and actions aimed at promoting health from an early age.

An International Study of Movement Behaviours in the Early Years – the SUNRISE Study includes 64 countries that are geographically and culturally diverse and represent a broad range of economies as classified by the World Bank [31]. The primary aim of the SUNRISE Study is to monitor the proportion of 3- to 4-year-old children who meet the WHO global guidelines for movement behaviours for the early years in the participating countries [2]. In addition, the SUNRISE Study aims to determine if these proportions differ by sex or urban/rural location, and between countries with different levels of human and economic development. Secondary aims include monitoring the prevalence of overweight and obesity among the children as well as their cognitive and motor development. Further, the study identifies factors at different socioecological levels associated with movement behaviours among young children. These examined correlates/determinants of movement behaviours may differ between countries involved in the study. This article describes the protocol of the SUNRISE Study conducted in Finland.

The SUNRISE Finland Study aims to examine what proportion of 3- to 4-year-old children living in Finland meet the WHO global guidelines on 24-hour movement behaviours, and how that proportion and children’s motor and cognitive skills compare with children from other countries involved in the SUNRISE Study. We also aim to identify potential factors associated with children’s movement behaviours, focusing on family socioeconomic factors, residential environmental features (e.g. living in urban or rural environments), and mental health and wellbeing of two caregivers. The SUNRISE Finland Study examines factors associated with children’s movement behaviours at the society, family and individual levels. This will provide valuable insight into possible determinants of these behaviours and identify those children who are not meeting the movement guidelines. In addition, our study examines the associations between children’s movement behaviours, motor and cognitive skills, adiposity, and psychosocial wellbeing. Further, we aim to establish a longitudinal cohort of families who participate in the study and conduct follow-ups to examine, for example whether movement behaviours change and predict health, weight, movement behaviours, motor and cognitive skills and psychological wellbeing of the children in the future.

## Methods

### Study design

This project is part of the SUNRISE Study, which is an international cross-sectional study including 64 countries from each of the four levels of the United Nations Human Development Index (Low, Lower-Middle, Upper-Middle, and High) [31]. For the main study, each country recruits 1,000 children and their caregivers. The SUNRISE Finland Study includes the child measurements (accelerometers, anthropometrics, motor and cognitive skills) and the Parent Questionnaire included in the international SUNRISE Study [31]. In addition, we added the SUNRISE Finland Questionnaire on sociodemographic factors, parental movement and health behaviours, and parental and child mental health symptoms. In addition to the first caregiver, we also invite a second caregiver or other adult in child’s life to participate in the study by completing the SUNRISE Finland Questionnaire. We also measure children’s waist circumference as an additional measure. Detailed objective data on residential environmental features, which are not included in the international SUNRISE study protocol, will be collected utilizing Geographic Information System (GIS). Furthermore, we aim to establish a longitudinal cohort of the SUNRISE Finland’s participants and conduct follow-ups in the future.

### Participants and recruitment

**Fig. 1:**
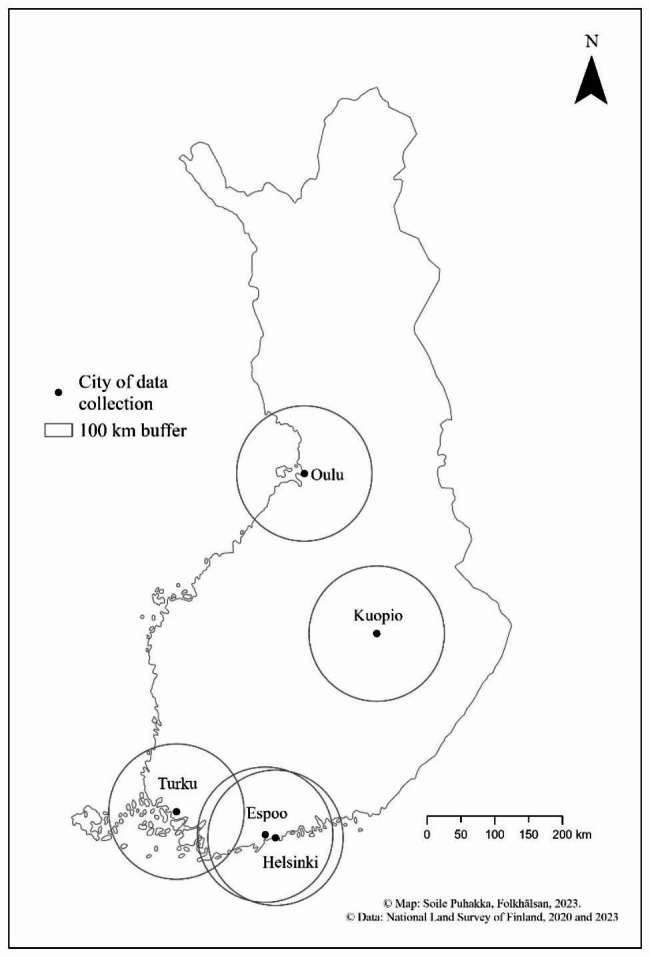
Five data collection cities in the SUNRISE Finland Study. 100 km buffers refer to areas from which the rural daycare centres where selected using the Urban–Rural Classification by the Finnish Environment Institute

The aim is to recruit at least 1,000 children and their parents/caregivers from early childhood education and care (ECEC) centres situated in different parts of the country to represent children living in geographically and socioeconomically differing areas in Finland. The initial aim was to recruit c. 250 children in each four data collection areas: in the cities of Helsinki, Turku, Kuopio and Oulu and the surrounding rural areas of each city (50% in urban and 50% in rural areas). However, a fifth data collection area was later added to the protocol: Espoo and the surrounding rural areas. Espoo is the second biggest city in Finland after the capital Helsinki. Helsinki and Espoo both belong to Uusimaa region, which is the most densely populated area in the country. A larger proportion of the Finnish population lives in Southern Finland compared to Northern Finland. Figure 1 shows the final five data collection areas in Finland.

Data will be collected in clusters; one ECEC centre is one cluster. First, ECEC centres will be recruited by emails and phone calls to ECEC directors in the communities and/or directors of the centres. Then, information letters and invitations to participate in the study will be send to parents through the centres in electronic and/or paper form, depending on each centre’s preference. All eligible children (aged 3.0 to 4.9 years) in the selected centres and one of their caregivers as well as another adult in the child’s life (parent/caregiver/grandparent etc.) will be invited to participate. The only exclusion criterion for the study is if a child is unable to wear an accelerometer. Before and during recruitment, we advertise the study to ECEC centres and parents by writing press releases and stories in local newspapers (and other media), posting about the study in social media groups and with localized Facebook ads, and bringing/sending posters and flyers (with QR codes to information videos and consent forms) to ECEC centres. Some cities’ communication and sports services departments help in advertising our study. We also give presentations about the study in ECEC director meetings when possible. The marketing methods may vary between cities and rural towns because, for example, possibilities to get stories published in local newspapers and permissions to attend directors’ meetings differ between the regions. Our data collectors will also visit yards of some of the participating urban ECEC centres to inform about the study to parents in person, but this is not feasible in rural areas that are geographically further away. We will continue the recruitment until we have collected data from 1,000 children. Trained research staff will conduct children’s measurements in the ECEC centres.

A pilot study was conducted between January and May 2022. Altogether 58 children from 12 Folkhälsan’s and five municipal ECEC centres in Southern Finland participated. The pilot study showed the study protocol was suitable for Finnish environment. We conducted two focus group interviews with five parents who participated in the study with their child. According to the interviews, the children mostly liked participating in the study measurements at the daycare centre and wearing “the superhero belts and watches”, which was how our data collectors called the accelerometers when giving those to children. The Axivity accelerometer’s rubber wrist bands were too loose for the wrists of some children. Therefore, for the main study, we decided to use fabric wrist bands that are more suitable for children’s small wrists. Similar fabric wrist bands were proven feasible among preschool-aged children in the JOYPAM project [32]. Fifty (86%) of caregivers completed the Parent Questionnaire in an electronic form. To increase the response rate, we decided to offer an option to complete the consent form and the questionnaires also on paper in the main study. Parents in the focus group considered most of the questions included in the questionnaires clear to understand and answer, even though some of the questions in the Parent Questionnaire did not suite very well for the Finnish environment or society. Training of the data collectors for the main study’s data collection in area 1 (Helsinki region) was conducted by the SUNRISE Study’s Principal Investigator professor Anthony Okely in May 2022 in Helsinki. Some of the same data collectors will be involved in data collection in areas 2, 3, 4 and 5, and the SUNRISE Finland research group will train new data collectors in each area.

### Measures

#### Society-related factors

**Fig. 2:**
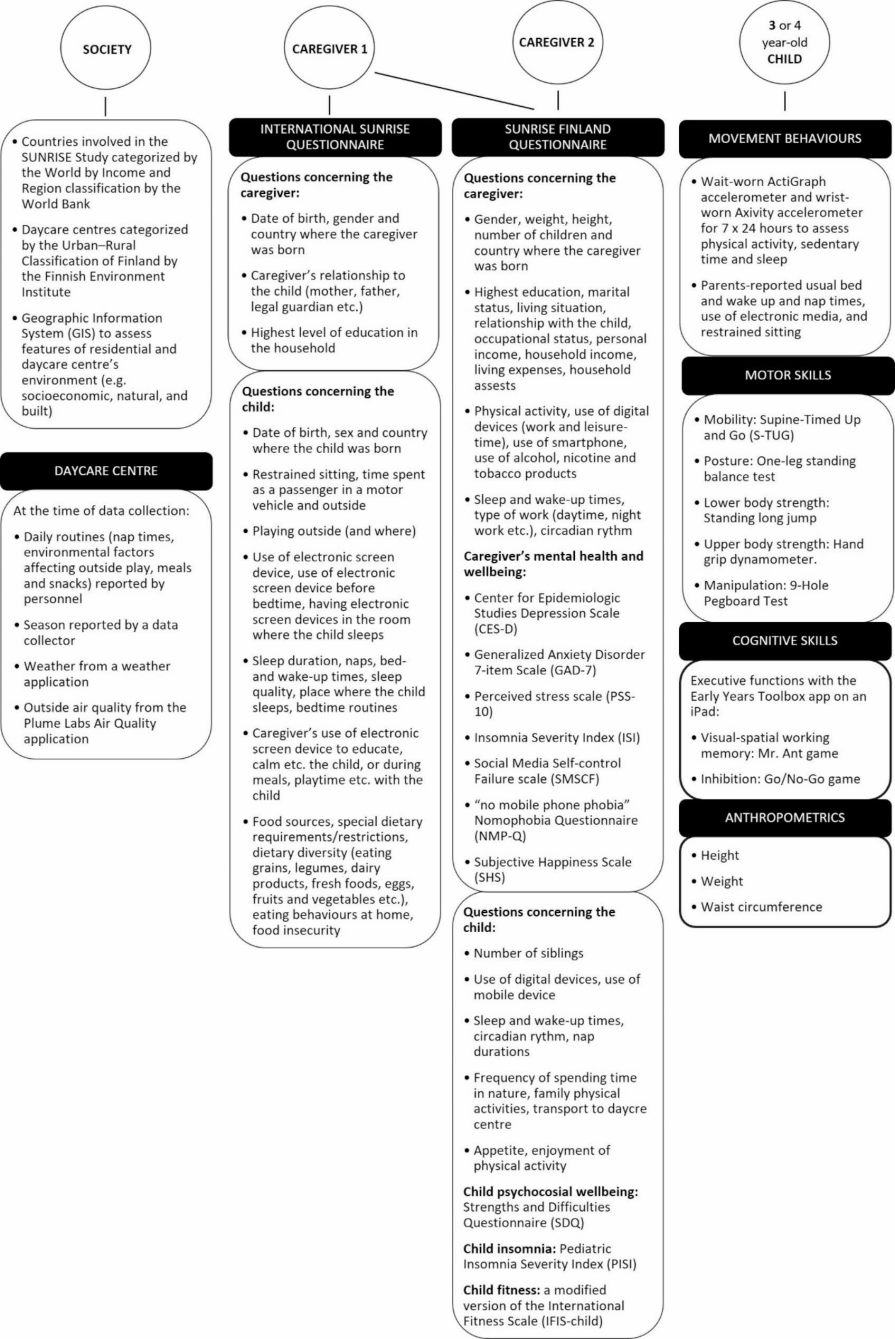
Summary of measures in the cross-sectional SUNRISE Finland Study

Figure 2 summarizes the measures included in the SUNRISE Finland Study. The countries that have been chosen for the SUNRISE Study are geographically and culturally diverse and represent a broad range of economies as classified by the World Bank. The ECEC centres will be selected from both urban and rural districts across Finland (in five data collection areas) to compare children living in different locations. Rural daycare centres will be selected in c. 100 km buffer from each data collection cities. GIS and the 2018 Urban–rural spatial classification of Finland provided by the Finnish Environment Institute (SYKE) will be used to select the urban and rural daycare centres based on their addresses [33]. The classification is based on several statistical features such as population, standard industrial classification of the workforce, CORINE Land Cover [SYKE], commuting, potential accessibility, and area density of buildings. The classification consists of two main regional classes: urban areas (inner urban area, outer urban area, peri-urban area) and rural areas (rural area close to urban areas, local center in rural areas, rural heartland area, and sparsely populated area). We will not recruit daycare centres in peri-urban area because those areas are a mixture of urban and rural areas. We will also examine rural and urban environments in more detail by collecting ECEC centres’ and participants’ addresses to geocode and calculate environmental variables by using GIS. GIS is a tool for gathering, analyzing and mapping data based on location provided, and can be used to assess the quantitative features of the environment, such as socioeconomic features, greenspace, or built environment. For example, a circular buffer with a certain km radius can be fixed around each participant’s residency or ECEC centre’s location to represent the nearby environment, as used in previous studies [34–36].

#### Children’s 24-hour movement behaviours

We will ask children to wear an ActiGraph accelerometry (wGT3X-BT or GT9X) on their waist and an Axivity AX3 accelerometry on their non-dominant wrist continuously (including sleep) for a minimum of 7 × 24 h to assess weekday and weekend PA, sedentary behaviour and sleep. The accelerometers will be positioned with an adjustable elastic waistband and adjustable wristband. The waist-worn accelerometer is not waterproof and should be taken off when bathing, showering, or in a sauna. The wrist-worn accelerometer is waterproof and should be taken off only in sauna because of the high temperature. The waist-worn ActiGraph accelerometer is the most widely used and extensively validated device for assessing PA, sedentary time and sleep in preschool-aged children [37, 38], however, wrist placement of an accelerometer has shown superior compliance compared to waist placement in school-aged children [39]. A wrist-worn Axivity accelerometer has been shown to be feasible to use among preschoolers, but further validation studies are needed to validate this device for movement behaviours in this age group [40]. We include both devices since a waist-worn ActiGraph is included in the international SUNRISE protocol, and a wrist-worn Axivity will provide additional information on acceptability and feasibility of accelerometer use among 3- to 4-year-old children. Data collectors will attach the monitors on children during other measurements in ECEC centres. Parents will receive information sheets on the use of accelerometers and will complete a monitor log during the seven days, in which they will report the time when the child goes to bed and wakes up, and whether the child wears the accelerometer when sleeping, and on which days the child was in the ECEC centre. In addition to accelerometers, movement behaviours will be reported by the parent/caregiver on behalf of the participating child with questions that were originally developed based on the recommendations for each behaviour guideline [41]. Parents will report their child’s usual bed and wake up and nap times (from which sleep-time is calculated), and use of electronic media and restrained sitting (to calculate sedentary screen time and restrained sitting). Parents will also report the use of screen-based devices before bedtime, the presence of devices in the room in which the child sleeps, and parental use of electronic media during everyday interactions with the child.

#### Child motor skills

Gross and fine motor skills will be measured via validated activities from the National Institutes of Health (NIH) Toolbox [42, 43].

##### Mobility and posture

Supine-Timed Up and Go (S-TUG). A line is marked 3 m from a wall. The child lies supine (on their back) with their feet (heels) on the line. On “go” the child is required to get up as quickly as possible, run and touch a target on the wall, and run back across the 3 m line.

##### Posture and balance

One-leg standing balance test. The child stands on each leg (the right leg first), with the arms held freely at the side of the body for up to 30 s.

##### Lower body explosive strength and mobility

Standing long jump. The child jumps as far as they can and lands on two feet.

##### Upper extremity strength

Hand grip dynamometer. The child squeezes the grip with each hand continuously with full force for at least 3 s without letting their arms touch their body.

##### Dexterity or manipulation

9-Hole Pegboard Test. The test assesses fine motor dexterity or manipulation. Dexterity is a central component of hand function and relates to both the speed and accuracy of hand movements. The protocol includes one practice and one timed trial with each hand.

The selected measures for child movement behaviours and motor skills have been proven feasible among 3- to 4-year-old children in the SUNRISE pilot studies and in other previous studies [44–48].

#### Child cognitive skills

The Early Years Toolbox app on an iPad will be used to assess executive functions. These toolbox measures have shown very good reliability, convergent validity with existing measures, and developmental sensitivity among preschoolers and early primary school students [49]. Children will play two brief validated games from the toolbox. The first game measures visual-spatial working memory – the amount of visual information that a child can concurrently coordinate in mind. The task includes a cartoon character Mr. Ant who have different colored dots placed in different locations on his body. The dots will disappear after a predetermined time and the child will be asked to touch the screen to recall the locations of dots. The second game is called Go/No-Go and measures inhibition – ability to control behavioural urges and impulses. The child will be asked to catch fish by tapping the screen of an iPad and to avoid the sharks by refraining from touching the screen whenever the shark appears.

#### Anthropometrics

Data collectors measure children’s height, weight, and waist circumference using WHO protocols [50]. From those, body mass index z-score and waist-to-height ratios will be calculated.

### Parent questionnaires

One of the child’s parents/caregivers will be asked to fill in the Parent Questionnaire included in the international SUNRISE study protocol [31]. In addition to assessing key sociodemographic factors and a child’s movement behaviours, the questionnaire assesses potential factors associated with a child’s movement behaviours. The content of the questionnaire is summarized in Fig. 2.

In addition to the SUNRISE Study’s Parent Questionnaire, we will ask two of the child’s parents/caregivers/adults in the child’s life to fill in the SUNRISE Finland Questionnaire. This questionnaire assesses sociodemographic and family-related factors (Fig. 2), such as living situation and property type with modified questions previously used in the Helsinki Health Study [51], occupational status with a question used in the FinHealth Study [52], personal and household income with questions used in the DAGIS Study [24, 53], household assets with a question from the Whitehall Study [54], and marital status as well as relationship to the child and living with the child with questions developed for the SUNRISE Finland Study.

The SUNRISE Finland Parent Questionnaire also assesses caregivers’ and children’s health behaviours: caregiver’s PA with questions previously used in the FinHealth and FINFIT Studies [52, 55], caregiver’s and child’s use of digital devices with questions developed for the SUNRISE Finland Study, caregiver’s logged smartphone and child’s logged mobile device use with modified questions used in the DigiConsumers research project [56], and circadian rhythm of the caregiver and the child with modified questions from the Morningness–eveningness questionnaire’s (MEQ) item 19 [57]. Caregiver’s use of nicotine and tobacco products will be assessed with modified questions used in the FinHeath Study [52] and the use of alcohol with the short AUDIT-3 questionnaire [58]. Frequency of spending time in nature will be assessed with modified questions used in the DAGIS study [24, 53], family PA and child’s enjoyment of PA with questions from the Skilled Kids study and the JOYPAM project [32, 59], child’s commute to the daycare centre with a question used in the JOYPAM project [32], and child’s appetite with a question from the Healthy Start project [60].

### Mental health and wellbeing

The following brief and validated self-report questionnaires will be used to assess the caregiver’s mental health symptoms and mental wellbeing.

#### Depression

The Center for Epidemiologic Studies Depression Scale (CES-D) measures depressive symptoms severity in the general population. CES-D provides cut-off scores (e.g. 16 or greater) with acceptable screening accuracy for clinical depression [61, 62], and has shown good psychometric properties [63].

#### Anxiety

The Generalized Anxiety Disorder 7-item Scale (GAD-7) measures symptoms of anxiety and is a validated and efficient tool for screening generalized anxiety disorder [64, 65].

#### Perceived stress

The Perceived stress scale (PSS-10) measures the degree to which situations in one’s life are appraised as stressful. PSS-10 has shown good internal consistency and good construct validity [66, 67].

#### Insomnia

The Insomnia Severity Index (ISI) measures perceived sleep difficulties. ISI has shown adequate internal consistency and reliability [68, 69].

#### Social media self-control failure

The Social Media Self-control Failure scale (SMSCF) has shown good internal consistency and test-retest reliability to assess social media self-control failure [70].

#### Nomophobia “no mobile phone phobia”

The Nomophobia Questionnaire (NMP-Q) measures the discomfort, nervousness or anxiety caused by being out of contact with a mobile phone. The NMP-Q has shown excellent internal consistency [71].

#### Happiness

The Subjective Happiness Scale (SHS) assesses the perception of general happiness. SHS features a good to excellent reliability, and validation studies support its use in assessing the construct of happiness [72, 73].

#### Child psychosocial wellbeing

Parents will be asked to report their child’s prosocial behaviour and psychological wellbeing with the Strengths and Difficulties Questionnaire (SDQ). SDQ has shown good internal consistency, inter-rater and cross-informant agreements and test-retest reliability [74, 75]. This questionnaire was added to the study protocol in the middle of the data collection, so the data will not be available from all participants.

#### Child insomnia

A parent-reported Pediatric Insomnia Severity Index (PISI) assesses a child’s insomnia symptoms and has shown preliminary reliability and validity in pediatric populations [76, 77].

#### Child physical fitness

A parent-reported modified version of the International Fitness Scale (IFIS) [78] estimates a child’s fitness. The modified version for preschoolers has shown acceptable test-retest reliability but poor criterion validity for assessing physical fitness in Spanish 3- to 5-year-old children [79].

### Other measures

Data collectors will gather information on aspects of ECEC centres’ daily routine by asking the ECEC centres’ personnel. In addition, data collectors will report in which season the data were collected, how was the weather and air quality during the data collection day (using Plume Labs Air Quality and weather applications), and on possible meals and snacks provided by the centre. We added the weather application measure to the protocol because the weather in Finland varies a lot on a daily and especially on a seasonal basis.

### Power and sample size estimation

Sample size estimation for the SUNRISE main studies was calculated based on data and response rates obtained from SUNRISE pilot studies from 17 countries. These pilot studies showed that the proportions of children meeting all components of the WHO movement behaviour guidelines varied across countries from 2.3 to 42.7% with a mean country proportion of 21.0%, with proportions differing within many countries when comparing rural with urban areas. The mean absolute difference in the proportion between rural and urban was 9.6% [31]. The power calculation is based on achieving 80% power and a 5% significance level for each participating country with the assumption of 21% of children meeting all guidelines and detecting a difference of 9.6% in either direction (two-sided) between urban and rural with an equal allocation to both rural and urban settings. This will provide an effect size of 0.23 (small effect), and results in a sample size of 558 children per country. When assuming a response rate of 76%, based on an average response rate in the 16 pilot studies, the required sample size increases to 734 children. The sampling of the main study will be partly based on cluster sampling (ECEC centres). The intra-class correlation (ICC) was estimated for each country, but the country-specific ICC estimates vary widely and are unreliable because of low pilot sample sizes in each country. Therefore, countries were combined and the ICC was estimated after controlling for country-specific effects. The resulting ICC estimate was zero, which might be, however, too optimistic (resulting in smaller sample size) and resulting in too low power, if the ICC is in fact positive in the main study. To be cautious, an ICC estimate of 0.022 from the PATH-ABC study was used [80]. With this value and assuming that 20 children (on average) out of 25 (on average) recruited children participate per daycare centre, the sample size of 735 is further increased to approximately 1000. In addition, this sample size is large enough to have a margin of error of at most 5% for a 95% confidence interval for the proportion of meeting all guidelines for each country and for both rural and urban populations when centred around 21% [31]. Thus, the sample size needed in each country was set to 1000 children.

### Data processing and analysis

Data included in the international SUNRISE Study (accelerometer data, motor and cognitive skills data, and data from the SUNRISE Parent Questionnaire) will be processed at the SUNRISE Coordinating Center at Early Start, University of Wollongong, Australia. The proportion of children meeting the WHO 24-hour movement behaviour guidelines in participating countries will be determined based on data from ActiGraph accelerometers (total PA, moderate- to vigorous-intensity PA and sleep) and from the SUNRISE Parent Questionnaire (sedentary screen time and restrained sitting).

For the international SUNRISE Study, the ActiGraph accelerometer data will be cleaned and scored using an automated script developed in R (version 4.2.1). First, the raw accelerometer data files (.gt3x) will be converted into counts per second format using the ‘ActivityCounts’ package [81]. The data will then be collapsed into 60-second and 15-second epochs using the ‘PhysicalActivity’ package for the analysis of sleep and PA, respectively. Sleep periods will be identified using the decision-tree-based algorithm in the ‘PhysActBedRest’ package [82] based on the vertical axis data, which has shown good sensitivity (0.936), specificity (0.970) and accuracy (0.952) compared to visual identification of sleep periods among preschoolers. Sleep periods that occur between 10:00 AM to 7:00 PM will be categorized as daytime naps. Non-wear during sleep will be defined as at least 90 min of consecutive zero counts with up to two minutes of non-zero interruption [83], while non-wear during waking hours will be defined as ≥ 20 min of consecutive zero counts [84]. Time spent sedentary (< 200 counts/15-sec), in light-intensity PA (200–419 counts/15-sec), moderate-intensity PA (420–841 counts/15-sec), and vigorous-intensity PA (≥ 842 counts/15sec) during waking hours will be defined and calculated using the cut-points developed by Pate et al. [85, 86]. Data from partial monitoring days (i.e., < 24 h period), as well as days with less than 10 h of valid waking wear time and 160 min of total sleep periods will be considered invalid and excluded from the analysis [87, 88].

For the SUNRISE Finland Study, GIS variables (i.e., land use features) will be created using different spatial data sources using ArcGis Pro software (ESRI, Redlands, CA, USA). The most current spatial information will be linked to match the timing of the data collection. The quantitative variables will be calculated to represent participants’ residential environment and daycare centres’ environment, including nearby neighborhood. The final environmental data will consist of information, for example, on natural areas and area’s land use, road network, socioeconomy and services. The data calculations for the GIS variables will be conducted at Oulu Deaconess Institute Foundation, Department of Sports and Exercise Medicine, Finland.

The associations of socioeconomic factors, residential environmental features, and mental health and wellbeing of caregivers with children’s movement behaviours will be investigated using, for example, correlation coefficients, chi-square test, and linear, binomial logistic and multinominal logistic regression analyses as well as linear mixed models. Similar statistical methods will be used when examining the associations between children’s movement behaviours, motor and cognitive skills, adiposity, and psychosocial wellbeing.

Additional processing and analysis methods for the accelerometer and other data may be used by researchers who will utilize data collected for the SUNRISE Finland Study.

## Discussion

The SUNRISE Finland Study will provide information on what proportion of 3- to 4-year-old children living in Finland meet the WHO movement behaviour guidelines, and how that proportion and children’s motor and cognitive skills compare with children from other countries involved in the international SUNRISE Study. Information on factors associated with children’s movement behaviours, meeting the movement behavior guidelines, and motor and cognitive skills will also be reported. For example, we will study the relationship of caregiver-related factors, such as their mental health, with children’s movement behaviours. We will also examine features of built and natural environment possibly promoting or restricting PA of the children living in rural and urban environments.

The SUNRISE Finland Study is part of the international SUNRISE Study, which provides new knowledge on movement behaviours of young children around the world. Involvement in the study provides an opportunity for international collaboration related to children’s movement behaviour research. We are collaborating with four Finnish universities in data collection areas, and with other studies examining children’s movement behaviours in Finland. In addition to being part of the international SUNRISE Study and thus enabling the international comparisons for its part, the SUNRISE Finland Study provides additional novel research evidence on factors associated with movement behaviours among young children at individual, family and society levels. Our study includes measures, such as children’s waist circumference and psychosocial wellbeing, parental mental health, as well as the GIS method to examine environmental features, that are not included in the international SUNRISE study protocol. Children recruited for the study will represent children living in different parts of Finland, and the country specific results on factors associated with movement behaviours will be, at least partly, generalized to other high-income countries. The results of the project will be presented in scientific journals and conferences, as well as in the media to spread the results. This project is multidisciplinary and generates new information for the purpose of promoting healthy movement behaviours and equal opportunities for healthy growth for all children. The results of this project can be used for advocacy purposes to inform key stakeholders on the prevalence of and factors associated with movement behaviours at an early age, potentially driving changes in policies. The SUNRISE Finland Study contributes to promoting health in the Finnish society, as well as in promoting children’s health globally by being part of an important international study.

## Data Availability

Not applicable.

## Abbreviations

CES-D: Center for Epidemiologic Studies Depression Scale
GAD-7: Generalized Anxiety Disorder 7-item Scale
ECEC: Early childhood education and care
ICC: Intra-class correlation
IFIS-Child: International fitness scale for children
ISI: The Insomnia Severity Index
MEQ: Morningness–eveningness questionnaire
NIH: National Institutes of Health
NMP-Q: Nomophobia Questionnaire
PISI: Pediatric Insomnia Severity Index
PSS-10: Perceived stress scale
SDQ: Strengths and Difficulties Questionnaire
SYKE: Finnish Environment Institute
WHO: World Health Organization
